# Supplementary material for: Multiscale based nonlinear dynamics analysis of heart rate variability signals
Source: PLoS One. 2020 Dec 17;15(12):e0243441. doi: 10.1371/journal.pone.0243441 (PMC7746153; doi:10.1371/journal.pone.0243441)
Supplement: Attachment — Submitted filename: Response to Reviewers.pdf [file pone.0243441.s001.pdf]

Response to Reviewers

Reviewer #1 Suggestions and Comments

This paper discusses a multiscale based nonlinear dynamics analysis of heart rate variability signals. The presentation of the content is fine, and the data is well explained. However, it is unclear what is new here compared with the existing methods. In addition, the significance of the results is lacked. Therefore, I suggest to decline this paper for publication

- The contributions of this research are twofold.
    - The traditional acceleration change index studied the dynamics of heart rate variability (HRV) signals at single time scale. HRV signals are outcome of highly integrated systems operating at multiple time scales. Hence ACI may fail to extract correct but subtle information encoded in HRV signals genrated by these systems operating at multipe time scales. Healthy subjects reveal lower ACI values, whereas aging and disease is associated with increase in ACI. It is evident from Table 2 and Fig 3, ACI (TACI at $\tau = 1$) showed dynamically incorrect results for AF subjects (0.54±0.11) compared to NSR (0.57±0.09). Incopration of multiple time scales resulted in lower ACI for NSR subjects compared to both AF and CHF subjects revealing that dynamical route of disease is associated with increase of ACI.
    - The outcomes of our study show that classification ability of ACI is modest. Incoporating mutiple time scales have resulted in better speration between healthy and different pathological subjects at wide range of threshold values. Table 3 shows that highest classification between NSR Vs CHF and NSR Vs AF was obtained at scale 3, whereas, maximum sepration between pathological groups CHF Vs AF was obrained at scale 10. The comparison of ACI and MACI at optimal scale represented in terms of errorbar plot is shown in Figure 2 and in terms of area under ROC (AUR) is shown in Figure for depicting degree of sepration. The findings aver that along with specific MACI vaues,we need to take into account their dependence on time scale for better characterization of HRV signals of healthy and pathological subjects.

- To show the significane, the proposed method MACI is compared with two more recently proposed nonlinear techniques(Improved Multiscale Permutation Entropy (IMPE) and Multiscale Normalized Corrected Shannon Entropy (MNCSE)).

  - Shah, S.A.A., Habib, N., Nadeem, M.S.A., Alshdadi, A.A., Alqarni, M. and Aziz, W., (2020). Extraction of Dynamical Information and Classification of Heart Rate Variability Signals Using Scale Based Permutation Entropy Measures. TRAITEMENT DU SIGNAL, 37(3), pp.355-365.
  - Awan, I., Aziz, W., Shah, I.H., Habib, N., Alowibdi, J.S., Saeed, S., Nadeem, M.S.A. and Shah, S.A.A., (2018). Studying the dynamics of interbeat interval time series of healthy and congestive heart failure subjects using scale based symbolic entropy analysis. PloS one, 13(5), p.e0196823

- The performance parameters clearly demonstrated that proposed MACI provided better separation between the groups than traditional ACI, IMPE and MNCSE measures.

<u>Reviewer #2 Suggestions and Comments</u>

The proposed method of using time evolution of heart rate variability captured by the so called Multi-scale based Acceleration Change Index for heart abnormality detection is very interesting. I have the following suggestions before the publication.

- Thank you for encouraging remarks and suggestions. We have incorporated the suggestions to improve manuscript.

The abstract is too long. I would suggest to make the abstract concise and remove the . For instance, the importance of heart rhythm monitoring for early diagnosis of disease using ECG signals is a well-known fact and that part can be summarize.

- Abstract is summarized as per requirement.

I suggest you add a more comprehensive References. For instance there exist recent works on predictive analysis of ECG signals that solve similar problems

- We have incorporated this suggestion and added more comprehensive references.

Use consistent format for acronyms. For instance lowercase first letter is used for "differentiated time series (DNN)" while uppercase first letter is used for "Acceleration Change Index (ACI)"

- Thanks for this suggestion. The suggestion has been incorporated

Please clarify why the sign information (the slope of acceleration) is sufficient for some diagnosis cases, as it is known that there are many abnormalities like "supraventicular arrhythmia" that can be captured by analysing the morphology of the ECG signal

- In this study, we investigated dynamics of heart rate variability signals to assess cardiac autonomic control of nervous system. Thus we need not to consider the morphology of the ECG signal. Instead, we extracted RR-intervals (interbeat intervals) from ECG signal and used MACI, which is an extension of ACI for assessing dynamical fluctuations of healthy and different pathological subjects. The sign change in the time series occurs when an increasing interval is immediately followed by the decreasing interval and vice versa. ACI increases only when a local maximum is followed by local minimum and vice versa, which detects the presence of very high frequency content in the HRV signal. Thus, sign information is sufficient for investigating the dynamical fluctuations of the heart, which assist the clinicians to asses cardiac autonomic control and its variations occuring due to aging and disease.

The results show a strong improvement for MACI vs ACI analysis in terms of the area under the ROC curve. I would encourage you compare your method to some existing methods in addition to the internal comparison between the ACI and MCI approaches.

- Suggestion has been incorporated and Compared MACI with Two more recently proposed nonlinear techniques (Improved Multiscale Permutation Entropy (IMPE) and Multiscale Normalized Corrected Shannon Entropy (MNCSE))

    - Shah, S.A.A., Habib, N., Nadeem, M.S.A., Alshdadi, A.A., Alqarni, M. and Aziz, W., (**2020**). Extraction of Dynamical Information and Classification of Heart Rate Variability Signals Using Scale Based Permutation Entropy Measures. TRAITEMENT DU SIGNAL, 37(3), pp.355-365.

    - Awan, I., Aziz, W., Shah, I.H., Habib, N., Alowibdi, J.S., Saeed, S., Nadeem, M.S.A. and Shah, S.A.A., (**2018)**. Studying the dynamics of interbeat interval time series of healthy and congestive heart failure subjects using scale based symbolic entropy analysis. PloS one, 13(5), p.e0196823.

The performance parameters clearly demonstrated that proposed MACI provided better separation between the groups than traditional ACI, IMPE and MNCSE measures.

Reviewer #3 Suggestions and Comments

Based on an existing index Acceleration Change Index (ACI) for describing heart rate variability (HRV), this paper proposes to feed coarse-grained HRV data to ACI. The so-called multiscale ACI (MACI) seems to improve the performance of ACI in distinguishing between healthy and pathologic subjects. However, more validations are needed. Moreover, comparison to other methods is missing despite so many existing methods for this purpose.

- Suggestion has been incorporated and Compared MACI with Two more recently proposed nonlinear techniques (Improved Multiscale Permutation Entropy (IMPE) and Multiscale Normalized Corrected Shannon Entropy (MNCSE))

  - Shah, S.A.A., Habib, N., Nadeem, M.S.A., Alshdadi, A.A., Alqarni, M. and Aziz, W., (**2020**). Extraction of Dynamical Information and Classification of Heart Rate Variability Signals Using Scale Based Permutation Entropy Measures. TRAITEMENT DU SIGNAL, 37(3), pp.355-365.

  - Awan, I., Aziz, W., Shah, I.H., Habib, N., Alowibdi, J.S., Saeed, S., Nadeem, M.S.A. and Shah, S.A.A., (**2018)**. Studying the dynamics of interbeat interval time series of healthy and congestive heart failure subjects using scale based symbolic entropy analysis. PloS one, 13(5), p.e0196823.

The performance parameters clearly demonstrated that proposed MACI provided better separation between the groups than traditional ACI, IMPE and MNCSE measures.

Specific comments:

1. The proposed approach is called multiscale ACI. However, in the end, only one scale is used. The current approach is more like an ACI on coarse-grained time series with a tuneable scale parameter. Can you use different scales at the same time to make this a real multiscale approach? A simple example would be just taking the average over a few τ values. Of course, linear or non-linear models can be trained using multiple scales but this will involve more validation such as training-testing splits.

  - The result shown in Table 2 show the results MACI at time scales 1 to 10, however, in Figure 3, we have shown the results ACI and MACI (at optimal scale) for comparing their performance to separate different groups. In this study, we have used standard multi-scaling procedure proposed by Costa et al [2], however several variations multi-scaling procedures exist, which were proposed to address shortcomings of multi-scaling procedure for handling short data. As we are using long term data, standard multi-scaling procedure serves this purpose.

2. Is the coarse-graining procedure stable? In other words, if the averaging brackets are switched one step to the right (e.g., deleting x1 in the time series), will the new index change a lot? A more detailed quantitative justification for this would be helpful.

- We have incorporated this suggestion. We have added Figure 7 to quantitatively justify that coarse graining procedure is stable by switching the averaging brackets one step right (1 to 5 steps) at threshold values 1 to 10. The overlap of ACI for all threshold values depict that coarse graining procedure is stable.

3. This is related to comment 1. In Fig. 2, how is the choice of whether to use more scale made? Are you merely iterating over a predefined set of τ values? Or is there any criteria to decide whether to try more τ values given the current computation results?

- As explained in response to comment 1, we used standard multiscaling procedure proposed by Costa et al [2]. It is evident from Table 2 and Table 3, MACI is significantly lower for NSR subjects compared to CHF and AF subjects at time scales 2 to 5 and at time scales 2 to 10 respectively. The significant difference between CHF and AF was observed at times scales 4 to 10. The highest separation between NSR vs CHF and NSR vs AF was obtained at time scale 3, whereas for NSR vs AF subjects maximum separation was obtained at time scale 10. Thus, we merely iterated time scale iterated time scale values from 1 to 10 to characterize the heart rate dynamics of healthy and pathological subjects. The findings aver that along with specific MACI vaues,we need to take into account their dependence on time scale for better characterization of HRV signals of healthy and pathological subjects.

4. In the applications, there is an optimal τ value of 3. It would be helpful to test whether this optimal value is consistent, e.g., by subsampling the datasets and see if 3 is still an optimal value.

- We have incorporated this suggestion by adding Fig 6, to study the variations in the MACI values for different signal lengths to classify NSR and CHF subjects. The findings show that optimal separation between NSR and CHF was found at time scale 3 for signal length >1000, and for signal lengths <=1000, time scale 2 provided maximum separation followed by the time scale 3. The results demonstrate that almost for all the signal lengths optimal time scale 3 is consistent for classifying NSR and CHF subjects.

  The optimal value of time scale revealing highest separation between NSR vs CHF and NSR vs AF was 3, whereas for NSR vs AF subjects optimal scale was 10. Thus optimal scale is different for separating NSR subject from pathological (NSR Vs CHF of NSR Vs AF) subjects compared to optimal scale for separating two pathological groups is not same. The findings aver that along with specific MACI vaues,we need to take into account their

dependence on time scale for better characterization of HRV signals of healthy and pathological subjects.

5. The performance advantage of the coarse-grained approach is clear, but there is little interpretation presented. Can you use some example time series and visualize the difference among ACI, MACI with optimal τ, MACI with suboptimal τ on the intermediate calculation results as described in the ACI algorithm.

We can visualize the difference between ACI, Optimal ACI and suboptimal ACI, however it results in three figures each with5 subplots. However, steps of ACI algorithm as detailed by Garcia-Gonzalez [16] will remain the same.

6. Due to the small data size, I recommend using some statistical tests to further support the performance improvements observed in Fig. 4 and 7.

The suggestion has been incorporated and following text is added in a new section Statistical Analysis and Evaluation Metrics.

- The analysis of variance (ANOVA) was used to analyse the differences among groups means and their concomitant measures for three or more groups. The one-way ANOVA is an omnibus approach, significant F statistic indicates that group means are statistically different, if any one of the groups is significantly different, but does not make paired comparison. The Bonferroni post-hoc test was used for multiple comparisons among the groups. The degree of separation among groups was quantified by using Area under receiver operator characteristic Curve (AUC) values (Mcneil & Hanley, 1984). The AUC is generally distinguished index for quantifying the level of separation between the groups. The AUC values show perfect separation of two groups at maximum value that is 1 and 0.5 value of AUC correspond to separation of groups by pure chance.

7. There are so many other indices for characterizing HRV, such as the ones listed in “Shaffer, Fred, and J. P. Ginsberg. "An overview of heart rate variability metrics and norms." Frontiers in public health 5 (2017): 258.” Comparison to the popular ones should be included. The paper that introduced ACI (Gonzalez et al 2003) did do some comparison to four other indices. But that comparison was on a different dataset and more methods have been proposed during the pase 17 years. Therefore, a fair comparison to other methods on the dataset used in this paper should be included.

- We have incorporated the suggestion and results are compared with other studies.

8. This is related to comment 7. In the first paragraph of “Materials and Methods”, the authors claimed that “no other index except ACI provide significant difference between the groups”. In the original publication which this paper heavily based on, the authors compared ACI with four

other indices and found that ACI is the best among the five. Thus, this statement in its current form is too strong and the context of Gonzalez et al (2003) should be included here.

- The suggestion has been incorporated and context of Gonzaez et al (2003) is included.

9. There are many grammar errors in the manuscript. I strongly recommend the authors to carefully proofread the manuscript.

- We thoroughly read the manuscript and rectified grammatical errors and typos.
